# 

**Published:** 1861-10

**Authors:** 


### CONTENTS.---OCTOBER, 1861.

### ORIGINAL CONTRIBUTIONS.

PAGE

ART. I. Lectures on Military Surgery. By E. ANDREWS, A. M., M. D., Professor of Surgery, etc. Lecture VI........ 513
ART. II. Rupture of the Uterus. By JAS. S. KING, M. D., of Lemont, Ill.,........ 520
ART. III. Strangulated Hernia. By W. A. GORDON, M. D., of Wausau, Wis.,........ 523
ART. IV. Report on the Sanitary Condition of the City of Chicago. Prepared for the Regular Meeting of the Chicago Medical Society, Sept. 20th, 1861. By N. S. DAVIS, M. D., Member of the Sanitary Committee,........ 528
ART. V. A Case of Periodical Strabismus. By F. N. SMITH, M. D., LaHarpe, Ill.,.... 532

### THE CLINIQUE.

CHICAGO MEDICAL AND SURGICAL DISPENSARY.—Service, Prof. E. ANDREWS, M. D., Attending Surgeon. Lithotomy;—Lecture and Operation........ 533

### CORRESPONDENCE.

"MILK-SICKNESS." Letter from JOHN A. KENNICOTT, M. D., The Grove, Ill,........ 538
From GEO. W. FISHER, Morris, Grundy Co., Ill.,........ 539

### SELECTIONS.

Hints to Army Surgeons. JACKSON,........ 542
On the Therapeutical Employment of the Oxide of Zinc. WATERMAN,........ 545
HAMMOND on Chancre,........ 548

### BOOK NOTICES.

A Treatise on Diseases of the Joints. By RICHARD BARWELL, F. R. C. S., etc. etc.,..... 551
Treatment of F.actures of Long Bones by Simple Extension. By JOHN SWINBURNE, M. D., etc.,........ 554
The Morbid Effects of the Retention in the Blood of the Elements of the Urinary Secretion. By WILLIAM WALLACE MORLAND, M. D., etc., etc.,........ 557
The Pathology and Treatment of Venereal Diseases; etc. By FREEMAN J. BUMSTEAD, M. D., etc.,........ 558
WYTHE'S Pocket Dose and Symptom Book....The Physician's Visiting List, for 1862,..... 559

### EDITORIAL.

Medical Societies........ 559
What Ails Them?........ 560
Mal-Practice Suit,........ 561
Demonstrator of Anatomy....Chicago Sanitary Commission,........ 562
Opening of the Medical Colleges in Chicago....Action of Opium on the Genito-Urinary Organs,........ 563
Books and Pamphlets Received,........ 564
SPECIAL SELECTIONS—Chlorate of Potash in Stomatitis Materna........ 564
United States Sanitary Commission in Illinois........ 565
Medicine and Surgery in the Army—Quinine as a Prophylactic; PIROGOFF'S Operation.... 567
Arsenious Acid a Substitute for Quinine....Industry of JOHN HUNTER........ 571
Causes of Mild Stomatitis in Infants....Measles Pre-disposing to Fevers....Effects of Climate on Northern and Southern Troops........ 572
RUSSELL on American Military Surgeons....Oil of Valerian in Typhus........ 573
Glycerole of Chlorate of Potash... Tincture of Digitalis in Delirium Tremens,........ 574

